# An integrated approach for trace detection of pollutants in water using polyelectrolyte functionalized magneto-plasmonic nanosorbents

**DOI:** 10.1038/s41598-019-56168-6

**Published:** 2019-12-23

**Authors:** Paula C. Pinheiro, Sara Fateixa, Ana L. Daniel-da-Silva, Tito Trindade

**Affiliations:** Department of Chemistry – CICECO Aveiro Institute of Materials, University of Aveiro, 3810-193 Aveiro, Portugal

**Keywords:** Materials chemistry, Nanoscale materials

## Abstract

Resistance of pathogenic micro-organisms to conventional antibiotics is an essential issue for public health. The presence of such pharmaceuticals in aquatic ecosystems has been of major concern for which remediation and ultra-sensitive monitoring methods have been proposed. A less explored strategy involves the application of multifunctional nanosorbents for the uptake and subsequent detection of vestigial contaminants. In this study, colloidal nanoparticles (NPs) of iron oxide and gold were encapsulated in multi-layers of a charged polyelectrolyte (PEI: polyethyleneimine), envisaging the effective capture of tetracycline (TC) and its subsequent detection by Surface Enhanced Raman Scattering (SERS). Adsorption studies were performed by varying operational parameters, such as the solution pH and contact time, in order to evaluate the performance of the nanosorbents for the uptake of TC from water. While the magnetic nanosorbents with an external PEI layer (Fe_3_O_4_@PEI and Fe_3_O_4_@PEI-Au@PEI particles) have shown better uptake efficiency for TC, these materials showed less SERS sensitivity than the Fe_3_O_4_@PEI- Au nanosorbents, whose SERS sensitivity for TC in water has reached the limit of detection of 10 nM. Thus, this study highlights the potential of such magneto-plasmonic nanosorbents as multi-functional platforms for targeting specific contaminants in water, by taking into consideration both functionalities investigated: the removal by adsorption and the SERS detection across the nanosorbents’ surfaces.

## Introduction

Tetracyclines comprise compounds of a widely used class of antibiotics, exhibiting broad-spectrum antimicrobial activity and as such are often used in human therapy and in the livestock industry^1^. These antibiotic compounds are released into municipal wastewater due to incomplete metabolism in humans or due to inadequate disposal of unused antibiotics. In fact, it was found that about 70% of TC is mainly excreted through urine and feces in an unaltered form after its administration^2^. A consequence of such discharges into water is that the detection of TC in animal products and in aquatic environments has been reported at levels that range from ng/L up to μg/L^2–5^. Although the risk posed by the release of antibiotics into the environment has not been fully assessed yet, it is now accepted that in long term might cause serious threat to human health, namely because contributes for bacteria drug-resistance. Thus, innovative water-monitoring processes are needed for such type of contaminants that might control and mitigate their negative impact. In this context, the on-site application of colloidal nanosorbents seems attractive because besides removal of vestigial antibiotics from water it might allow *in situ* detection. This prospective application relies on the development of effective multifunctional nanosorbents, such as magnetic and SERS active colloidal nanoparticles (NPs)^6^.

SERS is a surface sensitive vibrational spectroscopic method that in certain cases allows the analysis of trace amounts of a chemical compound whose molecules are close or chemisorbed to solid surfaces, typically of Au and Ag^7–11^. In particular, stronger SERS signals have been observed for adsorbates placed in between clustered metal NPs, which have been associated to enhanced local electrical fields generated in the interparticle nanojunctions or at the apex features of anisotropic nanostructures, often referred as plasmonic hotspots^12–15^. Such features have been explored with great success in several applications, which include the trace detection of pollutants in aqueous systems^16–27^.

Colloidal nanosorbents offer advantages as compared to conventional adsorbents, mainly due to the high specific surface area, tunable particle size distribution, and ability for surface functionalization aiming the capture of target pollutants^28^. In particular, the use of magnetic nanosorbents has gained recent attention because it allows the pre-concentration of trace analytes by collecting the suspended particulates (i.e. nanosorbents) via an applied magnetic gradient^29–36^. In this context, magnetite (Fe_3_O_4_) has been regarded as an elective material due to the high magnetization saturation, which allows fast separation from the aqueous environment, and because it is relatively cheap and innocuous for the aquatic eco-systems^37–39^. Our interest in this field, prompted us to develop magnetic nanosorbents for SERS detection of TC. A first attempt involved the use of Fe_3_O_4_ decorated with star shaped colloidal Au NPs^40^. While these systems have shown great potential in terms of SERS detection, the results also indicated the need for improvements mainly concerning the long term colloidal stability that allows adsorption studies for this analyte.

This challenging task was explored in this research by investigating the use of surface functionalized magneto-plasmonic nanosorbents, comprising Fe_3_O_4_ loaded with colloidal Au NPs, wrapped with polyelectrolyte macromolecules. We hypothesized that by using this type of hybrid nanostructures, it will be possible to take advantage of synergies provided by the different functionalities of the constituent materials (Au, Fe_3_O_4_ and PEI), namely for monitoring vestigial TC in water. Thereby, the adsorption behavior of TC molecules onto the magnetic nanosorbents and consequently their SERS detection are described here for the first time, aiming to evaluate also the role of the polyelectrolyte molecules adsorbed on the nanosorbents’ surface.

## Results

**Figure 1:**
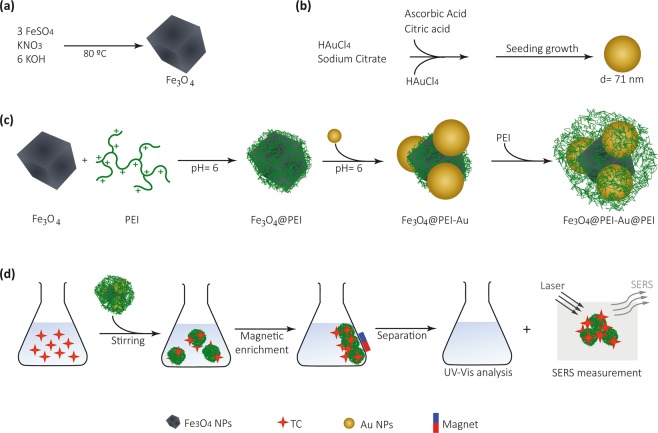
Schematic illustration of the: (**a**) synthetic route of cubic ferrimagnetic Fe_3_O_4_ NPs by oxidative hydrolysis under a nitrogen stream; (**b**) synthesis of spherical Au NPs by a seed growth method; (**c**) preparation of the magnetic-plasmonic polymer nanocomposite by the sequential addition of layers of PEI intercalated with Au colloid addition. At the end of each step, the nanosorbent was magnetically removed from the solution and washed thrice with deionized water; and (**d**) operating procedures for TC uptake by placing the nanosorbents in contact with aqueous solution of TC and then magnetically collected. The supernatant was analyzed using UV-Vis spectroscopy to assess the amount of TC molecules adsorbed on the multifunctional nanosorbents. The collected solid was placed in a glass slide and analyzed by SERS/Raman imaging using an excitation laser source at 633 nm.

This research reports hybrid inorganic-organic nanostructures composed of multilayers of Fe_3_O_4_ and Au NPs, alternately coated with a positively charged polyelectrolyte (PEI: polyethylenimine). Taking advantage of the presence of Au NPs in the multi-layered structures, Raman spectroscopic studies were then carried out aiming at the SERS detection of TC adsorbed in the nanosorbents. For sake of clarity, the general strategy followed in this work is outlined in Fig. 1.

### Synthesis and characterization of magneto-plasmonic nanosorbents

As a first step, colloidal cubic ferrimagnetic Fe_3_O_4_ NPs were prepared by partial oxidation of an aqueous ferrous salt using a mild oxidant, in alkaline conditions^41–43^. This method results in cubic shaped Fe_3_O_4_ particles about 80 nm in size, that are particularly useful for the magnetic separation envisaged in this work due to their high saturation magnetization (ca. 86 emu/g)^41,44^. These particles have been coated with the cationic branched-PEI polyelectrolyte that confers positive surface charge to the particles and will facilitate electrostatic interactions with citrate coated Au NPs via the protonated primary amine groups of PEI.

**Figure 2:**
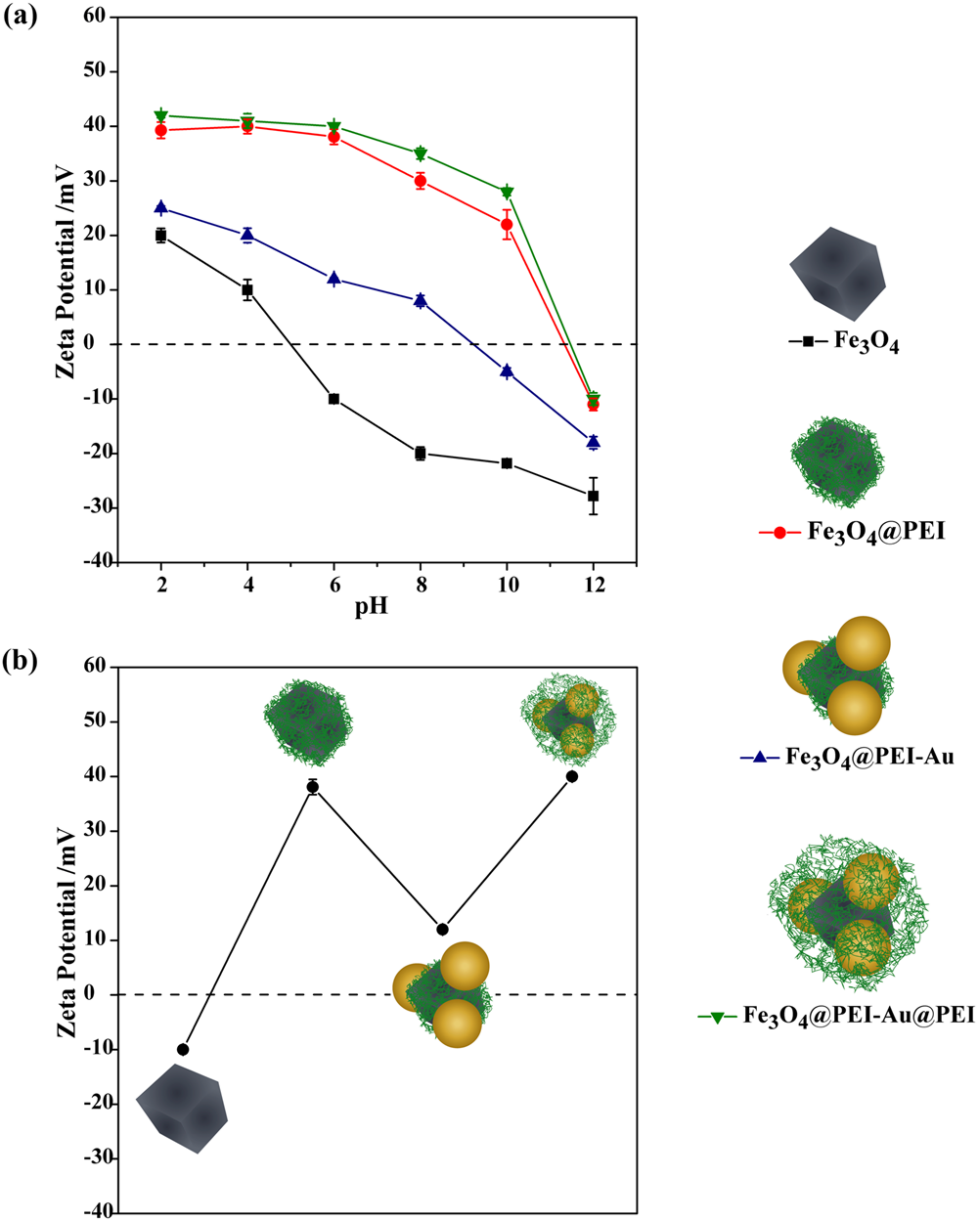
Zeta potential results for colloidal samples of Fe_3_O_4_ (squares); Fe_3_O_4_@PEI (circles); Fe_3_O_4_@PEI-Au (blue triangles) and Fe_3_O_4_@PEI-Au@PEI (green triangles), in function of pH **(a)**; zeta potential results for all nanosorbents at pH 6 **(b)**.

In order to obtain Au NPs with average dimensions (71 ± 5.1 nM) comparable to those of the Fe_3_O_4_ NPs, the Au colloids were prepared by using the seeded growth approach described by Ziegler and Eychmüller^45^. Moreover, such type of Au colloids have been reported to be highly SERS active under visible light excitation^46^. Finally the magneto-plasmonic assemblies have been treated again with PEI to provide a positive surface charge over the particles and to improve colloidal stability due to interparticle electrostatic repulsion. The overall assembly process was monitored by zeta potential titration of the colloids, as depicted in Fig. 2.

Figure 2 shows that at the selected working pH, the original negatively charged Fe_3_O_4_ particles became surface coated with PEI, which explains the increase of the zeta potential to +38.1 mV. Because the citrate coated Au NPs have a negative surface charge (−35.1 mV), these particles were attached electrostatically onto the positively charged surface of Fe_3_O_4_@PEI NPs, leading to a decrease on the zeta potential to +12.1 mV, at the same pH. The final Fe_3_O_4_@PEI-Au@PEI colloidal particles are positively charged due to another adsorption step with PEI, which compose the surface outer layers, because the pK_a_ value for the most basic (primary) amine group of PEI is 9.5, i.e. superior to the pH used in these experiments^47^. Indeed, the zeta potential of the PEI-modified colloids (Fe_3_O_4_@PEI and Fe_3_O_4_@PEI-Au@PEI) is positive over a wider pH range and the point of zero charge was found at 11.4. This result confirms that PEI macromolecules are present in the outer layer, otherwise the surface of the Fe_3_O_4_@PEI-Au particles, i.e. without the last post-synthesis PEI treatment, would be negatively charged for pH values above 9 (Fig. 2).

Figure 3.1 shows representative TEM images of magneto-plasmonic assemblies that have been collected from the respective colloidal suspensions. The type of inorganic NPs that compose the assemblies are clearly identified by their distinct morphology. This is in line with the presence of hybrid nanostructures that result from the interaction between cubic shaped Fe_3_O_4_ and spheroidal Au NPs. Note that the magnified TEM images show a thin layer of a less dense material coating the Fe_3_O_4_@PEI-Au@PEI assemblies, which we ascribe to the presence of the cationic polyelectrolyte PEI at the particles’ surfaces.

**Figure 3:**
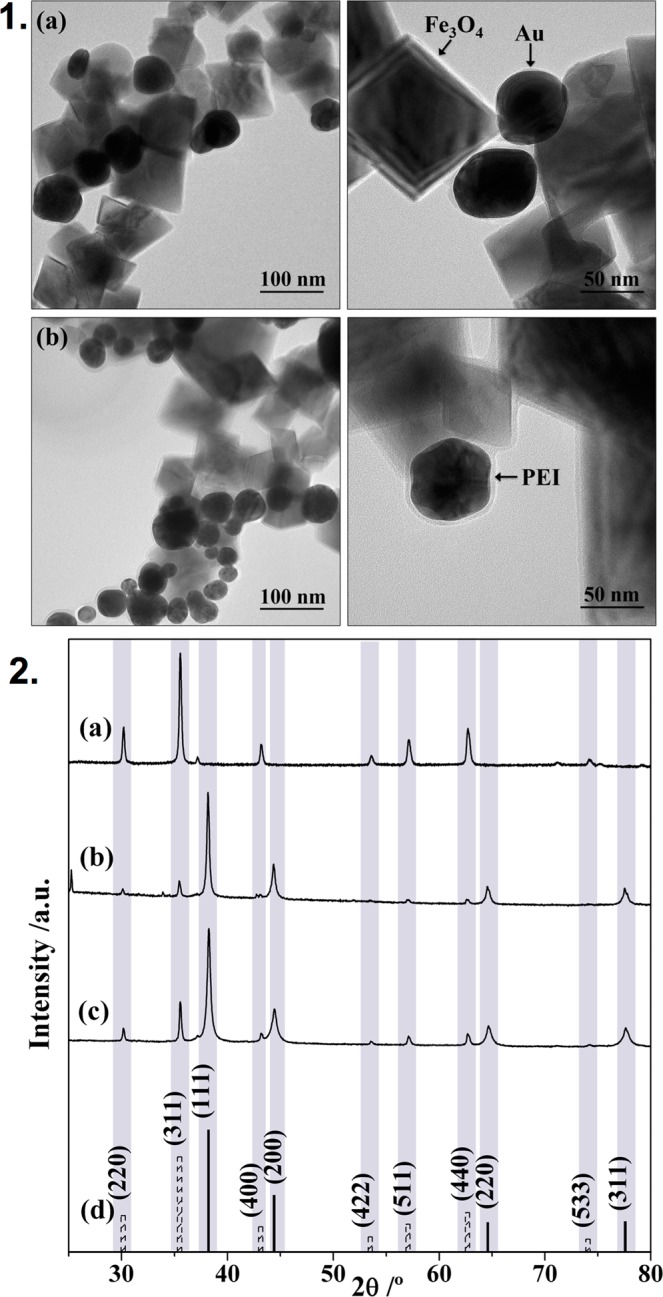
**1**. TEM images of the nanostructures: (**a**) Fe_3_O_4_@PEI-Au and (**b**) Fe_3_O_4_@PEI-Au@PEI; **2**. Powder XRD diffraction patterns of (**a**) Fe_3_O_4_@PEI; (**b**) Fe_3_O_4_@PEI- Au; (**c**) Fe_3_O_4_@PEI-Au@PEI; and (**d**) Face centered cubic Au (JCPDS Card No. 04-0784) (solid line) and Fe_3_O_4_ (JCPDS Card No. 19-0629) (dash line NPs).

The chemical identity of the inorganic nanophases shown by the TEM images was unequivocally demonstrated by comparing the powder XRD patterns for the single phases and the hybrid nanostructures. Hence, Figure 3.2 shows for the Fe_3_O_4_@PEI-Au@PEI sample a series of Bragg reflections (220), (311), (400), (422), (511), and (440), which are in accordance with the presence of the inverse spinel structure of magnetite (JCPDS file no. 19-0629). The four additional reflections (solid lines in Figure 3.2) are assigned to the (111), (200), (220) and (311) planes of Au NPs with face centered cubic (fcc) structure (JCPDS Card No. 04-0784).

Table 1 shows elemental microanalysis results that confirm the presence of PEI in the Fe_3_O_4_@PEI, Fe_3_O_4_@PEI-Au and Fe_3_O_4_@PEI-Au@PEI samples, namely by the C content that was found as 11.0 wt%, 9.2 wt% and 12.7 wt%, respectively. In addition, the results also indicate the presence of nitrogen in the hybrid particles due to the amine groups of PEI. Although the neat Fe_3_O_4_ particles also show C and H content, this is a negligible amount that is probably due to adventitious chemisorbed species.

**Table 1:** Elemental analysis for samples: Fe_3_O_4_; Fe_3_O_4_@PEI; Fe_3_O_4_@PEI-Au; Fe_3_O_4_@PEI- Au@PEI.

Sample	C(%)	H(%)	N(%)
Fe_3_O_4_	0.42	0.40	0.00
Fe_3_O_4_@PEI	11.00	6.30	2.00
Fe_3_O_4_@PEI-Au	9.20	5.90	2.30
Fe_3_O_4_@PEI-Au@PEI	12.70	7.10	3.90

The visible absorption spectra for the Au, Fe_3_O_4_@PEI-Au and Fe_3_O_4_@PEI-Au@PEI colloids are shown in Fig. S1 (Supplementary Information). As expected, the presence of Au NPs originates a localized surface plasmon resonance band (LSPR) at 542 nm, typical of quasi-spherical NPs with an average diameter of 70 nm. This optical feature for colloidal Au nanospheres is also observed in the visible spectrum of Fe_3_O_4_@PEI-Au sample but red-shifted to 578 nm, which can be explained by plasmonic coupling between neighboring Au NPs in the hybrid nanostructures^46^. This effect is somewhat diminished when a final PEI layer is added to the system (Fe_3_O_4_@PEI-Au@PEI nanocomposite) but still the LSPR is red shifted to 586 nm.

### Adsorption experiments of TC onto magneto-plasmonic nanosorbents

To have further insight about the effect of pH on the TC adsorption, the samples having PEI outer-layers were investigated as nanosorbents within the pH range 3–10 (Fig. S2). Both types of nanosorbents demonstrated to have a maximum percentage of TC removal (ca. 60%) in the pH range 5–8.

**Figure 4:**
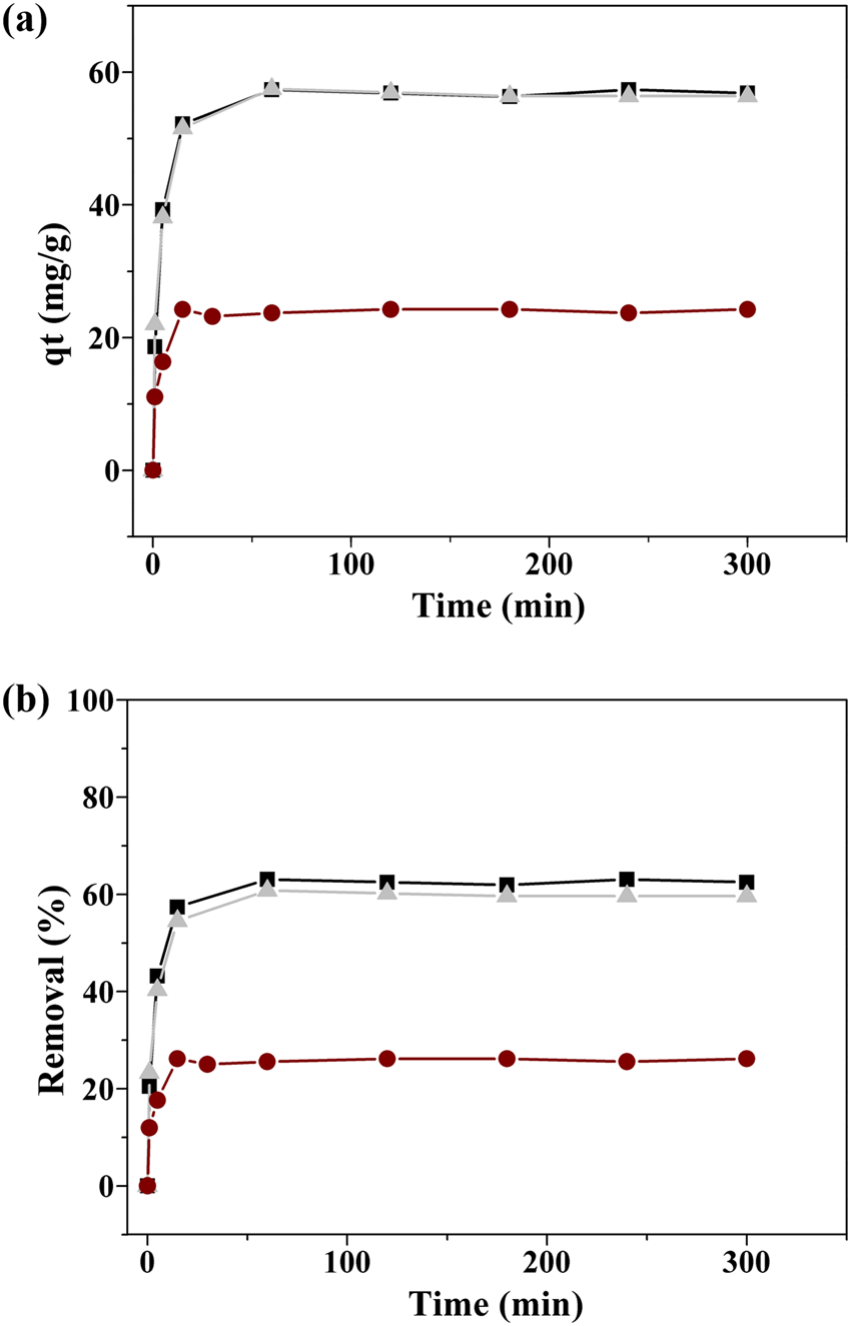
Time profile for (**a**) adsorption capacity and (**b**) removal percentage of TC (100 μM) from water using the nanosorbents: Fe_3_O_4_@PEI (squares), Fe_3_O_4_@PEI-Au (circles) and Fe_3_O_4_@PEI-Au@PEI (grey triangles), (pH 6, T = 25^o^C). Control experiments using tested solutions without nanosorbents were also carried out in parallel and revealed negligible loss of antibiotic during all the experiments.

The hybrid particles described above were investigated for the uptake of TC dissolved in aqueous solutions (100 μM), at pH ≈6, over a 13 h period. Nevertheless, rapid adsorption took place for all the nanosorbents tested with maximum adsorption capacity achieved at about 20–30 min contact time. The adsorption experiments in function of contact time for the several nanosorbents are presented in Fig. 4.

**Figure 5:**
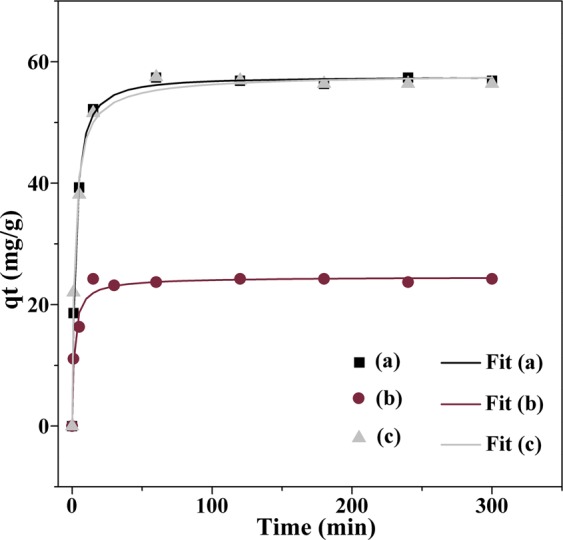
Modelling of adsorption kinetics of TC for (**a**) Fe_3_O_4_@PEI (square), (**b**) Fe_3_O_4_@PEI-Au (circle) and (**c**) Fe_3_O_4_@PEI-Au@PEI particles (grey triangle) using the general order kinetic equation.

The removal capacity observed for the magneto-plasmonic nanosorbents is attributed to the adsorption of TC molecules onto the particles’ surfaces. Four kinetic models commonly used in the study of solid-liquid adsorption processes were fitted to the experimental data: the pseudo-first-order, the pseudo-second-order and the general order equation that are kinetic models based on the chemical reaction, and the Elovich kinetic model that is an empirical model (model equations in the Supplementary Information). The kinetic parameters and the evaluation of the goodness of the fits, obtained by non-linear regression analysis, are reported in Table 2, and the kinetic fittings are shown in Fig. 5.

**Table 2:** Kinetic parameters estimated from pseudo 1^st^ order, pseudo 2^nd^ order, general order and Elovich models and evaluation of the respective fittings for initial TC concentration of 100 μM.

Model	Fe_3_O_4_@PEI	Fe_3_O_4_@PEI-Au	Fe_3_O_4_@PEI-Au@PEI	
pseudo 1^st^ order^a^	R^2^ (χ^2^)	0.9877 (2.4260)	0.9584 (2.8912)	0.9728 (5.3562)
	k_1_	0.2716	0.354	0.2899
	q_e_	56.24	23.73	55.75
pseudo 2^nd^ order^b^	R^2^ (χ^2^)	0.9979 (0.1457)	0.9801 (0.6726)	0.9936 (0.6434
	k_2_	0.0080	0.0281	0.0091
	q_e_	58.01	24.36	57.35
general order^c^	R^2^ (χ^2^)	0.9983 (0.1593)	0.9805 (0.6133)	0.9942 (0.4666)
	k_n_	0.0148	0.0148	0.0033
	q_e_	57.54	24.65	58.20
	n	1.83	2.23	2.27
Elovich model^d^	R^2^ (χ^2^)	0.9161 (11.4409)	0.9274 (2.7728)	0.9360 (8.1750)
	α	4334.8	3153.7	5050.8
	β	0.21	0.51	0.21

a: k_1_ (min^−1^); q_e_ (mg.g^−1^); b: k_2_ (g.mg^−1^.min^−1^); q_e_ (mg.g^−1^); *c*: k_*n*_ (min^−1^(g.mg^−1^)n^−1^)); q_e_ (mg.g^−1^); d: *α* (mg.g^−1^.min^−1^); *β* (g.mg^−1^).

Overall, the general order kinetic model provided the highest values of the coefficient of determination (R^2^) and the lowest values of the chi-squared value (χ^2^). The values were close to those obtained using the pseudo-second order model which is not surprising because the estimated order *n* is between 1.83 and 2.27, i.e., close to the second-order. It should be stressed out that when n = 2, the general order kinetic equation is the same as the pseudo-second order equation^48^. The residuals plots of the general order model (Supporting information, Fig. S3) show that the residuals are randomly distributed around the X-axis and are very close to zero, thus indicating that the model is suitable to describe the data. This is more evident for the materials Fe_3_O_4_@PEI and Fe_3_O_4_@PEI-Au, suggesting that chemisorption is the rate-controlling step for the adsorption process of TC molecules onto the NPs surfaces^48,49^. The model seems less suitable for the material Fe_3_O_4_@PEI-Au@PEI as the plot residual follows a pattern, with positive and negative residuals at short and longer contact times, respectively. It is possible that the extra PEI layer in this material affects the diffusion of TC molecules to the coated solid surface.

### SERS detection of TC using magneto-plasmonic nanosorbents

In a first step, the Raman spectra of Fe_3_O_4_@PEI-Au and Fe_3_O_4_@PEI-Au@PEI powders have been recorded in order to assess eventual spectroscopic features coming from the substrates. As shown in Fig. S4, these samples do not show Raman shifts in the spectral region under analysis, which favors their use as substrates for SERS analysis of TC. The same figure depicts the conventional Raman spectra of tetracycline hydrochloride powder and of aqueous solutions of different concentrations in TC (10^−1^ M and 100 μM). For the more diluted solutions no Raman bands were detected but the characteristic vibrational bands of TC (1619, 1447, 1315 and 1172 cm^−1^) are observed in the Raman spectrum of its solution with concentration 10^−1^ M. A more detailed assignment of the Raman active vibrational modes is provided in Table S1. The functional groups are given in parentheses whereby use is made of the numbering of the atoms as labeled in Fig. S5.

**Figure 6:**
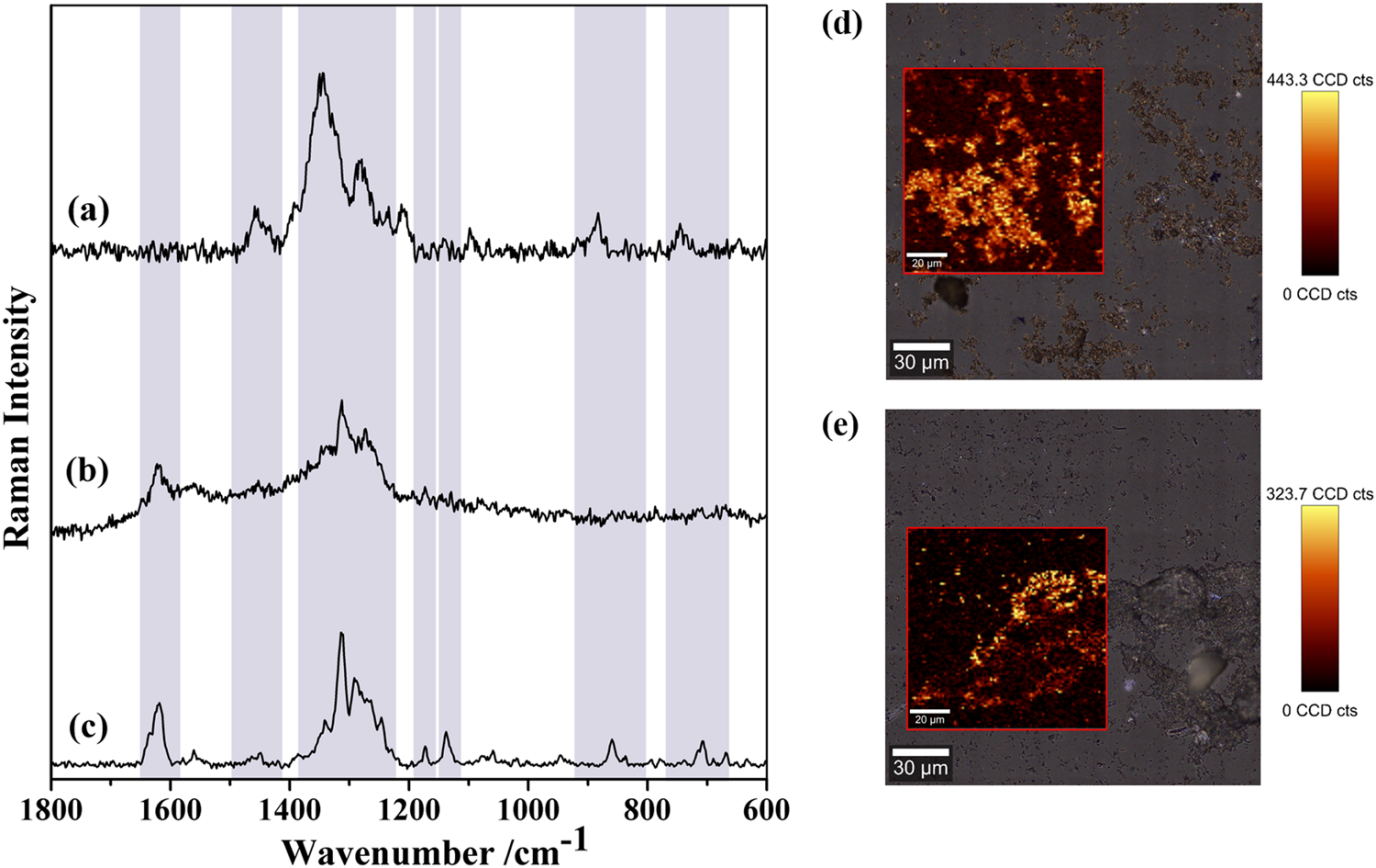
SERS spectra of TC (initial concentration 100 μM) adsorbed onto (**a**) Fe_3_O_4_@PEI-Au and (**b**) Fe_3_O_4_@PEI-Au@PEI nanosorbents (excitation line 633 nM); (**c**) conventional Raman spectrum of TC powder. Raman images obtained by integrating the band intensity at 1344 cm^−1^ of TC adsorbed onto **(d)** Fe_3_O_4_@PEI-Au and **(e)** Fe_3_O_4_@PEI-Au@PEI nanosorbents. The vertical bar shows the color profile in each image, with the relative intensity scale.

The SERS activity of the magneto-plasmonic nanosorbents was then evaluated by contacting the powders with TC aqueous solutions at the most diluted concentration (100 μM) and for which no bands were observed in the conventional Raman spectrum. As shown in Fig. 6, the Raman spectra of TC adsorbed onto Fe_3_O_4_@PEI-Au and Fe_3_O_4_@PEI-Au@PEI substrates show an enhancement of the Raman bands ascribed to TC molecules, as compared to the Raman spectrum of a TC solution analyzed in similar conditions. Control experiments performed by contacting Fe_3_O_4_ and Fe_3_O_4_@PEI powders with TC (100 μM) did not show Raman bands of TC, which indicates that the observed Raman signal enhancement is due to the presence of the Au NPs in the nanosorbents as consequence of a SERS effect. The Raman spectra of TC recorded by using both nanosorbents are similar (Fig. 6).

The limit of detection (LOD) of TC using the magneto-plasmonic nanocomposites was investigated by considering the lowest concentration at which the respective SERS spectrum was still observed. Thus, the LOD for TC using the Fe_3_O_4_@PEI- Au was 10 nM (initial concentration) and 10 μM using Fe_3_O_4_@PEI-Au@PEI.

**Figure 7:**
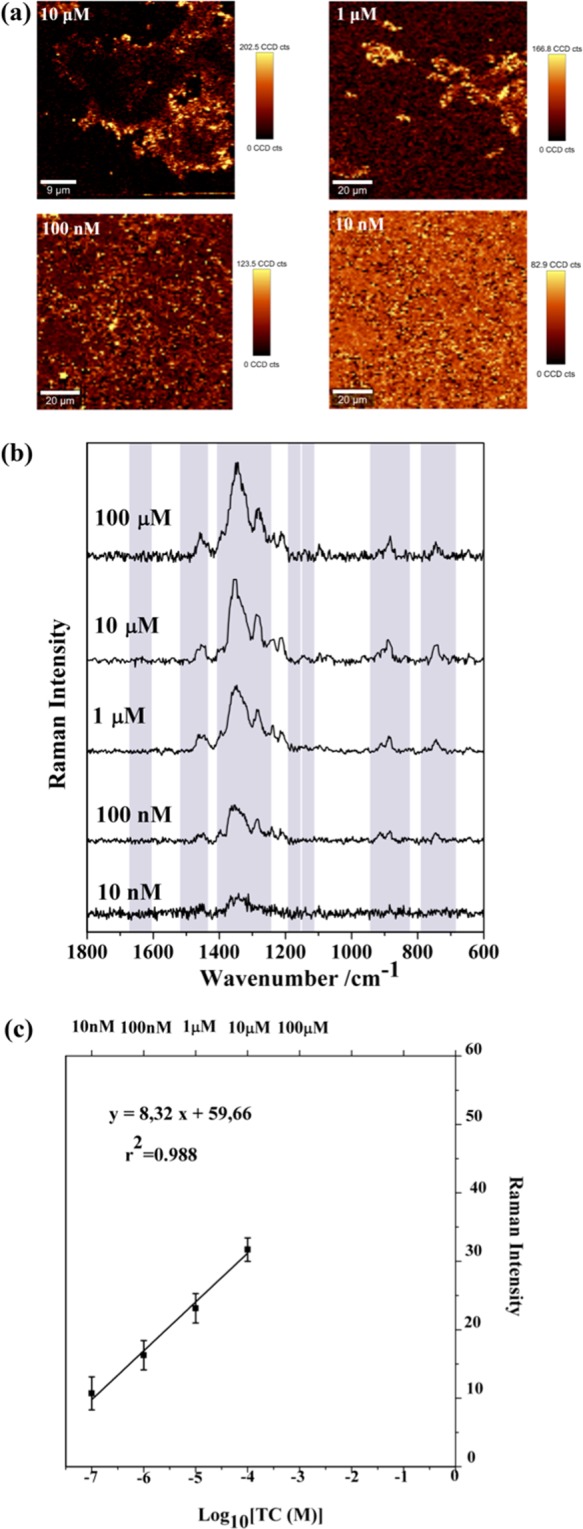
(**a**) Raman images obtained by using the integrated intensity of the band at 1344 cm^−1^ of TC for a concentration range of 100 μM to 10 nM and using Fe_3_O_4_@PEI-Au NPs as SERS substrate (excitation at 633 nm, 0.2 mW laser power, 150 points per line × 150 lines per image, 0.1 s). The vertical bar shows the color profile in each image, with the relative intensity scale; (**b**) SERS spectra of TC with different concentrations using Fe_3_O_4_@PEI-Au NPs as substrate obtained by the average of five spectra from the SERS analysis of two substrates; and (**c**) Relationship between the logarithmic initial concentrations of TC (100 μM to 100 nM) and SERS intensity at 1344 cm^−1^. Each square represents the average SERS intensity of the band at 1344 cm^−1^ and its standard deviation (vertical bars) acquired from five spectra on two substrates at different initial concentrations of TC.

The sensitivity of the Fe_3_O_4_@PEI-Au substrate was studied by performing SERS measurements using solutions with TC concentration varying from 100 μM to 10 nM. Figure 7 shows the spectra and the corresponding Raman images, which were obtained by monitoring the Raman band at 1344 cm^−1^. The Raman bands at 1315 and 1447 cm^−1^ in the spectrum of TC (10^−1^M, Fig. S4d) are observed but shifted to 1344 and 1455 cm^−1^, for example in the sample of TC 100 μM. Note that the most prominent band at 1344 cm^−1^ is still observed for a TC concentration as low as 10 nM. The plot of the peak intensity at 1344 cm^−1^ versus the logarithmic concentration of TC (nominal) originated a linear relation (R^2^ = 0.988) when the TC concentration ranges from 100 μM to 100 nM in water. The peak intensities of these spectra were obtained by using a peak fitting method. For each concentration, at least five spectra from two substrates were acquired and a statistical analysis of the results was conducted to obtain an intensity-concentration calibration curve (polynomial fitting), showing the mean and standard deviation of the peak intensities versus the logarithmic initial concentration of the analyte (Fig. 7c, Table S2). Although we are aware that only a fraction of the initial TC is adsorbed on the nanosorbents, these results are presented here to show that there is a linear response on the SERS band intensity (1344 cm^−1^) with the initial TC concentration. This suggests that the Fe_3_O_4_@PEI-Au nanosorbents afford promise as SERS platforms for sensitive and reproducible detection of TC. In this regards, the homogeneity of the substrates is a crucial feature to guarantee reproducibility of SERS analysis.

**Figure 8:**
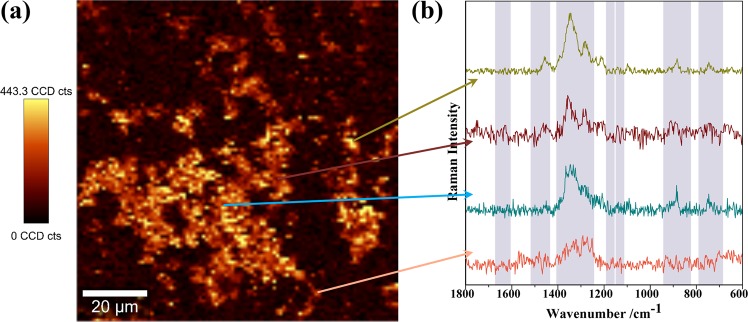
(**a**) Raman image obtained using the integrated intensity of the Raman band at 1344 cm^−1^ in the SERS spectrum of TC (100 μM) using the Fe_3_O_4_@PEI-Au as substrates (excitation at 633 nm, 0.2 mW laser power, 150 points per line × 150 lines per image, 0.1 s); and (**b**) selected SERS spectra of TC collected at different points as shown by the arrows (excitation at 633 nm, 0.2 mW laser power). The vertical bar shows the color profile with the relative intensity scale.

In order to investigate this issue, the spatial distribution of TC molecules over the surfaces of the Fe_3_O_4_@PEI-Au nanosorbents was investigated by Raman images constructed by integration of the TC band at 1344 cm^−1^ in several regions of the substrate, as shown in Fig. 8. The brighter colors in the Raman images indicate regions with more intense SERS signal, which might be due to the adsorption of TC molecules in regions of strong SERS activity, such as nanojunctions between Au NPs. The Raman images suggest homogeneous substrates at the micrometric scale by showing SERS signal ascribed to TC over the entire Fe_3_O_4_@PEI-Au surface.

## Discussion

In this research we have explored magneto-plasmonic nanosorbents comprising PEI multi-layers aiming at the removal of TC from water and its subsequent SERS detection. As such, a series of nanosorbents have been prepared and characterized for their chemical, morphological and surface characteristics using a plethora of techniques that include powder XRD, zeta potential measurements, TEM and electronic spectroscopy. Overall, the characterization performed has confirmed for all the hybrid nanosorbents the presence of cubic-type Fe_3_O_4_ particles coupled to Au nanospheres. The type of surface exposed to the water medium depended on the attachment of PEI layers at the surfaces of the particulates. Hence, the zeta potential measurements indicated that the nanosorbents having PEI outer-layers have the more positive surfaces. Indeed, the adsorption results show that these hybrid nanosorbents (Fe_3_O_4_@PEI and Fe_3_O_4_@PEI-Au@PEI) exhibit superior adsorption capacity as compared to those having Au surfaces exposed instead of PEI (Fe_3_O_4_@PEI-Au). The maximum percentage of TC removal (ca. 60%) was observed for the Fe_3_O_4_@PEI and Fe_3_O_4_@PEI-Au@PEI nanosorbents but much less (<25%) for the Fe_3_O_4_@PEI-Au samples. These results confirm that the use of PEI as a surface modifier impacts positively in the capture of TC molecules dissolved in water. Taking in consideration the role of the PEI molecules on the capture of TC molecules, we suggest that the adsorption of TC takes place mainly via electrostatic interactions between oppositely charged functional groups of TC and PEI.

Generally, four steps with distinct rates are associated to the solute transport in the solid-liquid adsorption process: the bulk diffusion, the film diffusion, the intraparticle diffusion and the physical or chemical adsorptive attachment onto the sorbent’s surface^50^. Herein four kinetic models were used to adjust the TC adsorption and the best fit was obtained with the general order kinetic model, which was selected based on the high coefficient of determination (R^2^) and low qui-square (χ^2^) values. These results suggest that the TC adsorptive attachment is the rate-limiting step^49,51^. Nevertheless for the sorbent Fe_3_O_4_@PEI-Au@PEI the residuals of this model were not randomly distributed around zero, which indicated poor fitting. A possible explanation is that the TC adsorption rate on this sorbent becomes diffusion-limited owing to the influence of the outer PEI layer in the diffusion of TC molecules.

The pH dependence behavior for the adsorption of TC can be understood by taking into consideration which ionized forms in the aqueous solution are able to interact with the PEI modified nanosorbents. Tetracycline is an amphoteric compound formed by a conjugated four-ring structure with various ionizable functional groups separated by a mostly flat hydrophobic structure of four six carbon rings for which three acidity constants have been defined (Fig. S5). The first deprotonation in TC involves the C1-C3 tricarbonyl system (pK_a_= 3.3) leading to the zwitterionic form of the neutral compound. The second pK_a_ at 7.7 is due to the deprotonation in C10-C12 phenolic diketone system and, above pH 9.7, TC exists in aqueous solution as a divalent anion due to the deprotonation of the C4-dimethylammonium group^52^.

As mentioned above, within the pH range under evaluation, the surfaces of Fe_3_O_4_@PEI and Fe_3_O_4_@PEI-Au@PEI are positively charged. Although the zwitterionic form of TC predominates at pH 3–7, still electrostatic interactions can occur between the negatively charged tricarbonyl methane and deprotonated ketophenolic hydroxyl groups and, the positively charged surfaces of the PEI modified nanosorbents. In addition, π-π stacking interactions involving adsorbed H_3_TC molecules may also favor the uptake of neutral TC in this pH window^53^. Thus, the maximum adsorption capacity of TC onto Fe_3_O_4_@PEI and Fe_3_O_4_@PEI-Au@PEI was found at pH 5–8 for which both types of interactions are favored. Although, both nanosorbents demonstrate similar efficacy for TC removal, the subsequent studies have been performed on Fe_3_O_4_@PEI-Au@PEI and Fe_3_O_4_@PEI-Au nanosorbents. Indeed, in these magneto-plasmonic nanosorbents, the presence of Au NPs is a requirement to observe enhancement of the Raman signal of the TC molecules that are adsorbed at the surface of the materials.

The Raman studies have shown similar SERS spectra for TC using either the Fe_3_O_4_@PEI-Au@PEI or Fe_3_O_4_@PEI-Au nanosorbents. In both cases, the most prominent bands at 747, 1344 and 1451 cm^−1^ correspond to normal modes involving CO stretching from ring B, C and D, thus suggesting the interaction of such functional groups with the Au surface^25,40,54^. However, using Fe_3_O_4_@PEI-Au as SERS substrate, the band assigned to the stretching and bending vibrational modes of both the amide-NH_2_ moiety and secondary amine of dimethylamino group (ring A) was not observed at 1619 cm^−1^ ^54^. The absence of this band suggests that the amine group in TC is not interacting with the AuNP because there is not a PEI layer that mediates such interaction. In fact, for the Fe_3_O_4_@PEI-Au nanosorbents because there is no PEI, the anionic groups of TC are free to interact directly with the Au surface. Despite the superior TC uptake performance of Fe_3_O_4_@PEI-Au@PEI as an adsorbent, the Fe_3_O_4_@PEI-Au particles have shown a lower LOD for TC in the SERS analysis that has been carried out. Also, the substrate Fe_3_O_4_@PEI-Au provided a better signal to noise ratio. This is a very important result, because the detection limit using Fe_3_O_4_@PEI-Au has the same order of magnitude as the maximum residue limit of 0.1 ppm (200 nM) in foods of animal origin, as defined by Commission Regulation (EU) 37/2010^55^. Taking into consideration a balance between the adsorption behavior and the SERS performance, the Fe_3_O_4_@PEI-Au materials appear as the most promising for the detection of TC in vestigial amounts, whose quantification is not trivial at such concentration limits. We propose two possible effects to explain these results; on one hand, the presence of an additional layer of PEI on the Fe_3_O_4_@PEI-Au@PEI particles can limit diffusion and subsequent interaction of the TC molecules with the Au NPs surface; and on the other hand, for the Fe_3_O_4_@PEI-Au@PEI composite, the TC molecules will interact preferentially with PEI rather than with Au NPs. The washing process before the SERS analysis can also have some influence because it can promote the release of the TC molecules that are interacting with PEI. All these effects can contribute for lower performance of the Fe_3_O_4_@PEI-Au@PEI particles as SERS substrates for the detection of TC. In fact, similar results were reported by Wang *et al*. but using instead Fe_3_O_4_@Ag microspheres coated with PEI^56^. The authors carried out a systematic study on the dependence of the Raman signal with the PEI layer thickness and have observed a decrease of the Raman signal with the increase of the PEI layer thickness, because fewer molecules can diffuse through the polymer and interact with the Ag surface. Additionally, they found that for an analyte interacting electrostatically with PEI, the Raman signal decreased by increasing the number of washed steps before SERS analysis. The principal contribution for the adsorption of TC onto Fe_3_O_4_@PEI-Au@PEI material was reported as the electrostatic interaction between negatively charged groups of TC molecules and PEI-amine groups. Conversely, the interaction between Fe_3_O_4_@PEI-Au and TC molecules could be explained by a combination of electrostatic interactions and covalent type/inner sphere type interactions^57–62^.

## Conclusion

Magneto-plasmonic nanosorbents based on Fe_3_O_4_ and Au encapsulated within a positively charged polyelectrolyte (polyethyleneimine) were successfully prepared. The ability of the resulting nanostructures to capture TC and to act as SERS active platforms was evaluated. The Fe_3_O_4_@PEI and Fe_3_O_4_@PEI-Au@PEI demonstrated to be more efficient to capture TC than in the absence of a PEI outer shell. This improved efficiency might be due to the outer PEI layer that confers strong affinity for TC molecules in aqueous solutions at the working pH, namely through electrostatic interactions between negatively charged groups of TC and positively charged amine groups of PEI. However, this outer PEI shell also limits the diffusion of TC molecules to the Au surfaces, thus decreasing the SERS sensitivity. We have concluded that the nanosorbent Fe_3_O_4_@PEI-Au provided a good compromise between Raman signal to noise ratio and LOD for TC. This type of nanosorbent showed high SERS sensitivity for an initial concentration as low as 10 nM, which is inferior to the maximum residue limit of 0.1 ppm in foods of animal origin, as defined by Commission Regulation (EU).

Our proposed magneto-plasmonic nanosorbents have shown great potential for the uptake/detection of TC in water and could be a powerful tool for fast and sensitive broad-spectrum antibiotic detection. Moreover, further studies are required using real water samples as they are more complex matrices.

## Methods

### Materials

The following chemicals were used as purchased: ferrous sulfate heptahydrate (FeSO_4_·7H_2_O, >99%, Panreac), citric acid monohydrate (C_6_H_8_O_7_·H_2_O, ≥99%, Panreac), potassium nitrate (KNO_3_, >99%, Sigma-Aldrich), sodium citrate tribasic dihydrate (Na_3_C_6_H_5_O_7_·2H_2_O, 99%, Sigma-Aldrich), chloroauric acid trihydrate (HAuCl_4_.3H_2_O, ≥99.9%, Sigma-Aldrich), branched polyethyleneimine (PEI, Mw = 25,000), potassium hydroxide (KOH, >86%, Pronolab), sodium hydroxide (NaOH, >98%, Pronolab), ascorbic acid (C_6_H_8_O_6_, J. M. Vaz Pereira), hydrochloric acid (HCl, ≥37%, Fluka), tetracycline hydrocloride (TC, C_22_H_24_N_2_O_8_, Sigma-Aldrich). All the solutions were freshly prepared using ultrapure water (18.2 mΩ·cm^−1^). All chemicals were used as received without further purification.

### Preparation of spherical Gold nanoparticles (Au NPs) by a seeded growth method

Au NPs were synthetized using a seeded- growth method, in which Au NPs with different diameters can be obtained. This approach consists on the reduction of a gold salt (HAuCl_4_·3H_2_O) using an aqueous solution containing ascorbic acid and sodium citrate as reducing agents. In a first step, Au seed particles were synthetized by the citrate reduction method, by the addition of 2 mL of sodium citrate aqueous solution (1% w/v, containing 0.05% w/v citric acid) to a HAuCl_4_·3H_2_O aqueous solution (50 mL, 0.25 mM) previously brought to a rolling boil (90 °C) under vigorous stirring. The colloid was then refluxed for more 5 min and then cooled at room temperature. These Au seeds have an average diameter of 15 nm and were used to synthetize larger Au NPs (diameter of 70 nm) following the Ziegler and Eychmüller’s method^45^.

### Preparation of multifunctional nanosorbents

Cubic magnetite NPs were prepared by partial oxidative hydrolysis of FeSO_4_·7H_2_O under a N_2_ stream^41–43^. Briefly, a solution of ultra pure water (60 mL), already deoxygenated, containing KNO_3_ (0.016 mol, 1.62 g) and KOH (11.23 g) was submitted to a N_2_ stream and then added dropwise to an aqueous solution of Fe(II) (0.5 M), at 90 °C. A black precipitate was formed which was stirred over 1 h at 90 °C, then left overnight and washed with deionized water. In order to functionalize the magnetite particles with PEI (Fe_3_O_4_@PEI), 10 mg of NPs were added to a PEI solution (300 mg, 10 mL). The pH of solution was adjusted to 6, using HCl solution (0.1 M) and the solution was stirred over 1 h. Then, the suspended solid was collected magnetically and washed thrice with deionized water to remove the excess of polyelectrolyte. The particles (10 mg) were then added to the Au colloid (200 mL) and the suspension was kept overnight in a flask placed in a minirotator. The nanocomposite particles were collected magnetically using a NdFeB magnet followed by washing thrice with deionized water (Fe_3_O_4_@PEI- Au particles). Finally, the particles were resuspended in 10 mL aqueous solution of PEI (450 mg) for 1h at pH 6. The final particles, Fe_3_O_4_@PEI-Au@PEI, were washed with deionized water and separated from solution using a magnet.

### Adsorption experiments

The capability of the multifunctional nanosorbents to remove TC from water was investigated by performing adsorption experiments. These studies were carried out using glass flasks containing aqueous solutions of TC freshly prepared from the dilution of the corresponding stock solution until achieving the desired initial concentration. Solutions of HCl (0.1 M) and of NaOH (0.1 M) were used for pH adjustment of the solutions used in the adsorption studies at variable pH. The nanosorbents were weighted and added to TC aqueous solutions of variable concentration and stirred under isothermal conditions (25 ± 1 °C), using a rotator with a constant rotation speed of 30 rpm (Grant Bio PTR-25 360° Vertical Mini Rotator). This time was considered as the starting point for the TC removal experiments. For each essay, the TC aqueous solution was shaken continuously and aliquots were collected for analysis at different contact times. The nanosorbents were then collected magnetically from the suspension using a NdFeB magnet. The amount of TC molecules in the supernatant was determined spectrophotometrically by measuring the absorbance at 358 nm. Calibration curves were obtained at TC concentrations ranging from 10 μM to 100 nM.

The amount of TC molecules adsorbed into the particles at time t (q_t_ in mg/g) was calculated using Eq. (1), where C_0_ is the initial TC concentration (mg/L), C_t_ is the concentration at time t (mg/L) in solution, V is the total volume of TC solution (L) and m is the mass of the dry weight of the sorbent (g).

1 $${{\rm{q}}}_{{\rm{t}}}={({\rm{C}}}_{0}-{{\rm{C}}}_{{\rm{t}}})\times {\rm{V}}/{\rm{m}}$$

The removal percentage (*R*) of tetracycline was calculated using Eq. (2):

2 $${\rm{R}}={({\rm{C}}}_{0}-{{\rm{C}}}_{{\rm{t}}}{)/{\rm{C}}}_{0}\times 100$$

Control experiments for TC removal in the absence of magnetic particles were also performed for similar experimental conditions as described above.

### Effect of contact time

The time profile of TC adsorption using these nanosorbents was assessed in order to evaluate the kinetics of adsorption. Briefly, 5 mg of each sample (Fe_3_O_4_; Fe_3_O_4_@PEI; Fe_3_O_4_@PEI-Au and Fe_3_O_4_@PEI-Au@PEI) were added to 10 mL of TC solution (10 μM) with the pH adjusted to 6. The mixture was then stirred (at 25.0 ± 1.0 °C) and aliquots of 0.7 mL were taken over time. The amount of TC adsorbed onto the nanosorbents at each time interval (q_t_, mg/g) was calculated using Eq. (1) and plotted in function of time (t, min).

### The effect of pH

The pH solution was varied between 3 to 10, in order to study the influence of pH on the TC adsorption behavior. Hence, the pH of the solution (10 mL, TC 10 μM) was set at the required value by using an alkaline solution (NaOH, 0.1 M) or an acid solution (HCl, 0.1 M). Then 5 mg of the powdered sample (Fe_3_O_4_@PEI, Fe_3_O_4_@PEI- Au@PEI) were added to the TC solution, using similar experimental conditions as described above and for a contact time of 20 min. UV-Vis spectroscopy was used to determine the final concentration of TC. At the end of the adsorption studies, the pH of the suspension was measured again in order to check for any variation on the pH due to the addition of the multifunctional nanosorbents.

### SERS studies

The SERS sensitivity of the nanosorbents was evaluated by the addition of the magnetic-plasmonic powders (Fe_3_O_4_@PEI-Au@PEI and Fe_3_O_4_@PEI-Au, 0.25 mg) to aqueous solutions of TC (10 mL) with different concentrations (100 μM–10 nM). The as prepared suspensions were shaken during 20 minutes, using a mini-rotor, at room temperature (25 °C) to allow the TC solution to contact with the nanosorbents. The solid was then collected magnetically from the solution and washed twice with ultra-pure water. The nanosorbent was then placed on a glass slide for SERS analysis and let it dry at room temperature (25 °C). Control experiments were also performed using neat Fe_3_O_4_ and Fe_3_O_4_@ as SERS substrates. SERS measurements have been acquired in different areas of the nanosorbents in order to evaluate the reproducibility of the measurements. The SERS studies were complemented with Raman images of the nanosorbents tested.

### Instrumentation

UV-Vis spectroscopy was used to determine the TC concentration in the supernatant solutions using a Jasco U-560 UV- Vis spectrophotometer (deionized water as the reference). The zeta potential measurements were performed by using a Zetasizer Nanoseries instrument from Malvern Instruments. The samples were prepared in ultra pure water solution at variable pH, from 2 to 12. Solutions of NaOH (0.1 M) and HCl (0.1 M) were used to adjust the required pH. Transmission electron microscopy (TEM) images were acquired using a Hitachi H-9000 TEM microscope operating at 300 kV. The TEM samples were prepared by dropping an aliquot of the diluted colloids on a carbon-coated copper grid and let it dry in air. For the X-ray power diffraction (XRD) measurements, an aliquot of the aqueous suspension of the nanosorbents was placed on a Si holder and let it dry at room temperature. The XRD data were acquired using a PAN analytical Empyrean X-ray diffractometer equipped with Cu Kα. The Raman maps were acquired using a combined Raman-AFM-SNOM confocal microscope WITec alpha300 RAS+. The Raman maps were obtained by the scan of a surface area of 2500 μm^2^ and accumulating a complete Raman spectrum at each pixel (in total 22500 spectra), with a spatial resolution of approximately 0.33 μm. The integration of the absolute area underneath the TC characteristic band at 1344 cm^−1^ was used to establish the color intensity scale in the Raman map. A He:Ne laser operating at 633 nM was used as excitation source with the power set at 0.2 mW. A 100× objective was used to view the samples, and the integration time for each spectrum was 0.1s. The time required to achieve a Raman image was 44 min. The limit of detection (LOD) was determined by monitoring the most intense Raman band of TC for the most diluted solution. The average intensity was obtained at each concentration using 5 replicas and the generated calibration curves consisted of the average signal within the range of initial concentrations.

## Supplementary information

Supplementary Information
