# Supplementary material for: An integrated approach for trace detection of pollutants in water using polyelectrolyte functionalized magneto-plasmonic nanosorbents
Source: Sci Rep. 2019 Dec 23;9:19647. doi: 10.1038/s41598-019-56168-6 (PMC6928026; doi:10.1038/s41598-019-56168-6)
Supplement: Supplementary file 1 — Supplementary Information [file 41598_2019_56168_MOESM1_ESM.pdf]

Supplementary Information

An integrated approach for trace detection of pollutants in water using polyelectrolyte functionalized magneto-plasmonic nanosorbents

Paula C. Pinheiro[a], Sara Fateixa[a], Ana L. Daniel-da-Silva[a], Tito Trindade[a]*

[a]*Department of Chemistry – CICECO Aveiro Institute of Materials*

University of Aveiro, 3810-193 Aveiro, Portugal.

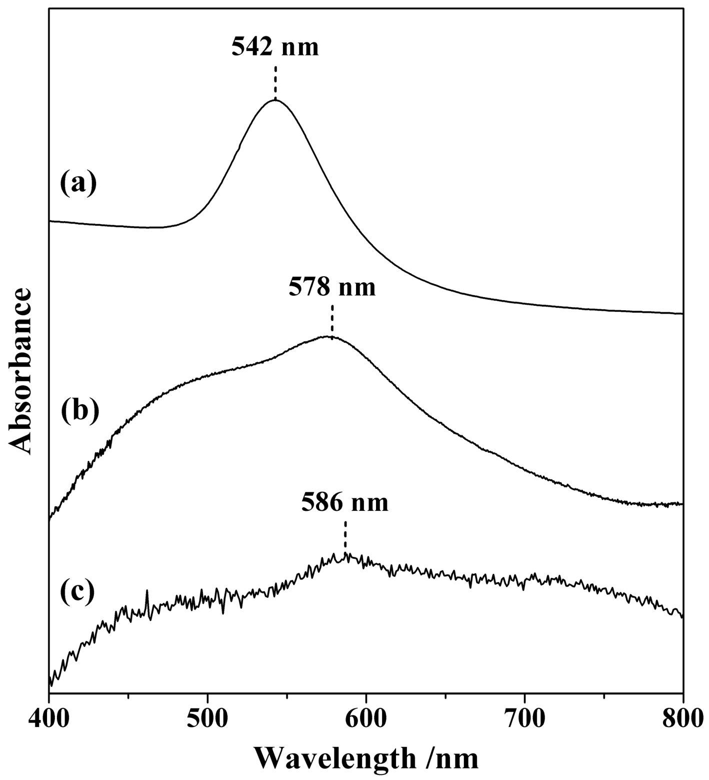

Figure S1. UV-Vis absorption spectra of (**a**) Au NPs (colloid); (**b**) Fe_3O_4@PEI-Au (powder); and (**c**) Fe_3O_4@PEI-Au@PEI (powder).

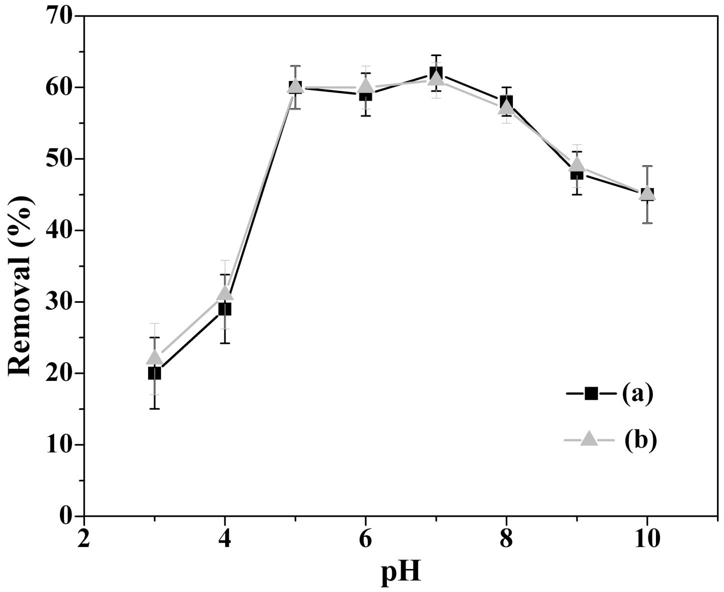

Figure S2. Effect of pH of the medium on the TC uptake, for an initial TC concentration of 100 μM and a contact time of 30 min, using (**a**) Fe_3O_4@PEI and (**b**) Fe_3O_4@PEI-Au@PEI particles as sorbent.

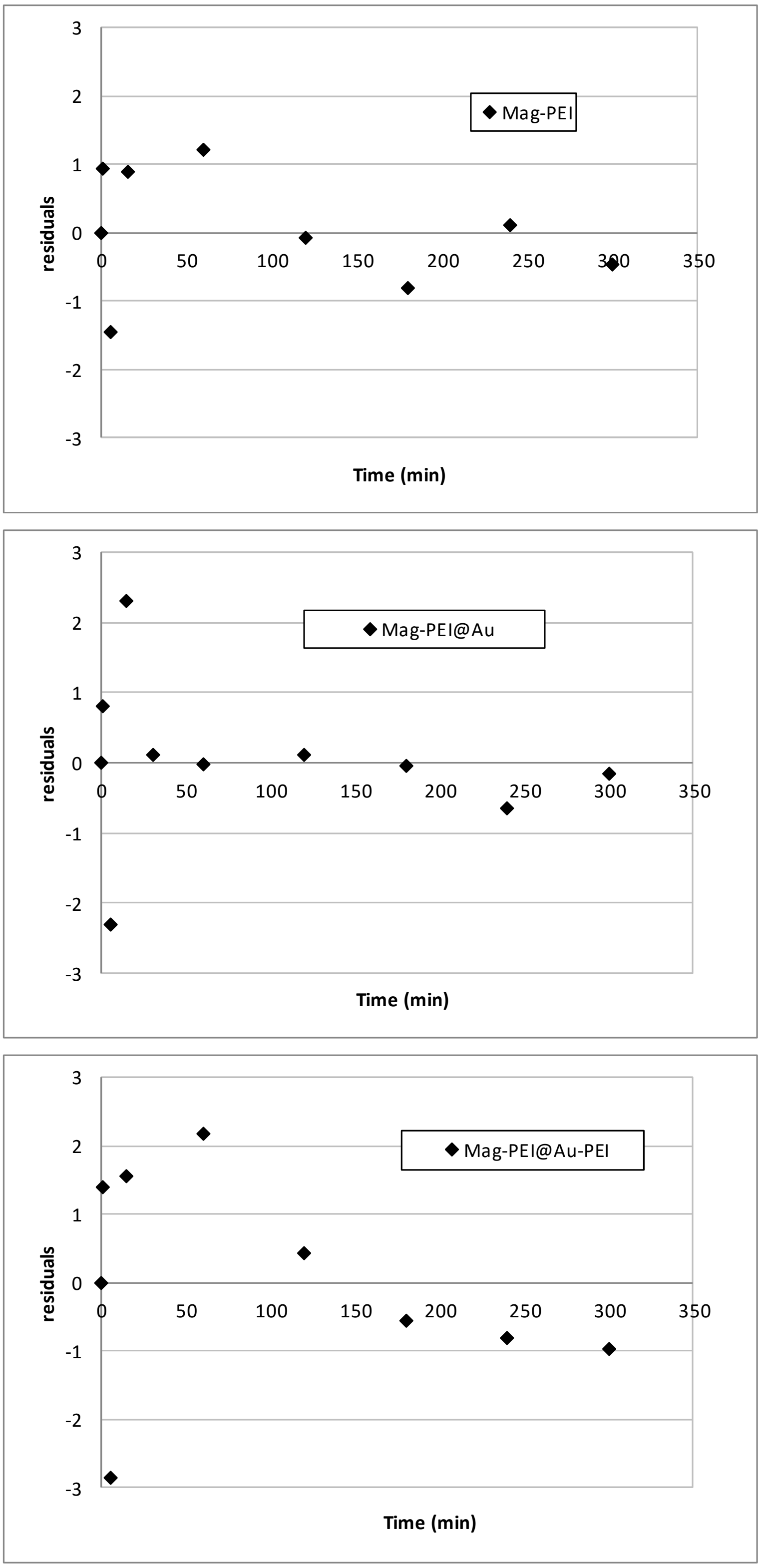

Figure S3. Residuals plot of the fittings employing the general-order kinetic equation.

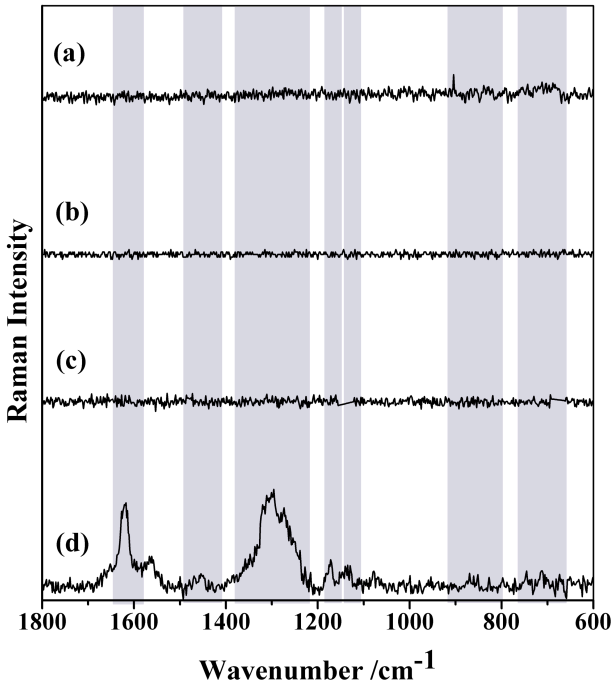

Fig. S4: Raman spectra: (a) Fe_3O_4@PEI-Au; (b) Fe_3O_4@PEI-Au@PEI; (c) tetracycline (100 μM); (d) tetracycline (10^{-1} M).

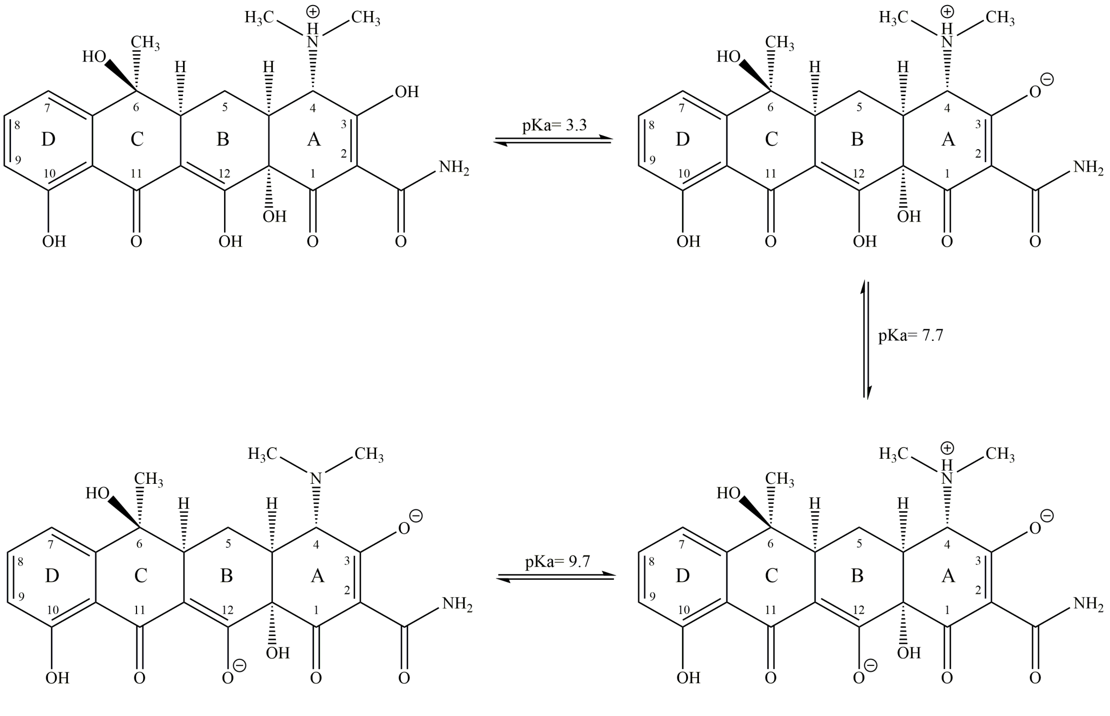

Fig. S5: Structural formula of tetracycline showing the ionisable forms and respective pKa values.[REF-1]

Table S1. Raman bands and respective assignments for the TC powder and TC adsorbed onto Fe_3O_4@PEI-Au used as SERS substrates [REF2-REF8].

Raman (cm^{-1})	SERS (cm^{-1})	Assignment
1619	-----	υ(CO1) + υ(amid-CO) + δ(amid-NH) + υ(CO3) + υ(C2-C3) + υ(OH10,12) + υ(amid-CO2)
1560	-----	u(C11O) + u(CC)D
1447	1459	δ(CH7,8,9) + δ(OH12) + υ(D) + υ(CO10,CO11,CO12) + δ(amin-CH_3) + δ($CH_3$6)
1315	1347	δ(OH10,12) + δ(CH4,4a,5,5a) + υ(C5aC11a) + υ(C1C2) + υ(C9C10,C10C10a,C10aC11) + υ(CO11,12) + υ(CO3) + δ(CH7,8,9)
1286	1283	δ(CH4,4a,5a) + δ(OH12) + δ(amid-NH) + υ(CO10) + υ(CO3) + υ(CH7,8.9) + υ(amid-NC) + υ(C4aC5) + υ(D)
1172	1175	υ(CO3)
1137	1133	υ(CO6) + υ(CO12)
859	888	δ(CC)A,B,C,D + υ(C15 O25)
707	747	ω(amid-NH), ν(CO6,12), γ(OH3,6,10,12,12a)

υ = stretching modes; δ = bending mode; ω, out-of-plane swing; γ, out-of-plane bending

Table S2. Values of the mean and standard deviation of the peak intensities of the Raman band at 1344 cm^{-1} using different concentrations of TC and the logarithmic initial concentration of the TC.

TC (M)	Log_{10}[TC (M)]	Raman Intensity
10^{-7}	-7	2.5±1.2
10^{-6}	-6	9±2.3
10^{-5}	-5	17±2.5
10^{-4}	-4	27±2.0

Adsorption Kinetic modeling

Non-linear form of the pseudo-first order (S1), pseudo-second order (S2) and general order (S3) equations and linear form of the Elovich (S4) kinetic model [REF9-REF13] $q_t = q_e(1 - e^{-k_1 t})$ (S1)

$$q_t = \frac{k_2 {q_e}^2 t}{1 + k_2 q_e t} \quad \text{(S2)}$$

$$q_t = q_c - \frac{q_c}{[k_n (q_c)^{n-1} t(n-1)+1]^{1/(n-1)}} \quad \text{(S3)}$$

$$q_t = \frac{1}{\beta}\ln(\alpha\beta) + \frac{1}{\beta}\ln(t) \quad \text{(S4)}$$

where, q_t and q_e ($mg.g^{-1}$) are the adsorption capacity at time t and equilibrium time, respectively; k_1 is the equilibrium rate constant of pseudo 1st order adsorption (min^{-1}), k_2 is the equilibrium rate constant of pseudo 2nd order adsorption ($g.mg^{-1}.min^{-1}$), k_n is the equilibrium rate constant of general order adsorption ($min^{-1}(g.mg^{-1})^{n-1}$)) and α and β are the Elovich coefficients and define the initial sorption rate ($mg.\ g^{-1}.min^{-1}$) and the desorption constant respectively ($g.mg^{-1}$).

The Elovich model is an empirical model that was firstly proposed by Roginsky and Zeldovich in 1934 [REF14] for the adsorption of carbon monoxide onto manganese dioxide. A simple and linear form of this model (equation S3) was later derivatized by Chien and Clayton [REF11]. This model has been used satisfactorily to describe chemisorption kinetics and is often valid for systems in which the adsorbing surface is energetically heterogeneous [REF 15].

The goodness of the fittings was determined based on the calculation of the correlation coefficient (R^2) (S4) and Chi-square test value (χ^2) (S5), expressed by the following equation respectively:

$$R^2 = 1 - \frac{\sum_{i=1}^{n}(y_i - \hat{y}_i)^2}{\sum_{i=1}^{n}(y_i - \bar{y})^2} \qquad \text{(S4)}$$

$$\chi^2 = \sum_{i=1}^{n} \frac{(y_i - \hat{y}_i)^2}{\hat{y}_i} \qquad \text{(S5)}$$

where y_i and $\hat{y}_i$ are the experimental and model predicted values respectively, $\bar{y}$ is the mean of the experimental data and n is the sample size.

References

[REF1] Gu, C. Karthikeyan, K. G. Interaction of tetracycline with aluminum and iron hydrous oxides. *Environ. Sci. Technol.* **39**, 2660-2667 (2016).

[REF2] Zhao, J. Liu, P. Yuan, H. Peng, Y. Hong, Q. & Liu, M. Rapid detection of tetracycline residues in duck meat using surface enhanced Raman spectroscopy. *J. Spectrosc.* **2016** (2016) Article ID 1845237

[REF3] Qu, L.-L. Liu, Y.-Y. Liu, M. Yang, G.-H. Li, D.-W. & Li, H. Highly reproducible Ag NPs/CNT-intercalated GO membranes for enrichment and SERS detection of antibiotics. *ACS Appl. Mater. Interfaces* **8,** 28180–28186 (2016).

[REF4] Dhakal, S. Chao, K. Huang, Q. Kim, M. Schmidt, W. Qin, J. & Broadhurst, V. A simple surface-enhanced Raman spectroscopic method for on-site screening of tetracycline residue in whole milk. *Sensors* **18,** 424 (2018).

[REF5] Filgueiras, A. L. Paschoal, D. Dos Santos, F. & Sant'Ana, A. C. Adsorption study of antibiotics on silver nanoparticle surfaces by surface-enhanced Raman scattering spectroscopy. *Spectrochim. Acta, Part A* **136,** 979–985 (2015).

[REF6] Jin, D. Bai, Y. Chen, H. Liu, S. Chen, N. Huang, J. Huang, S. & Chen, Z. SERS detection of expired tetracycline hydrochloride with an optical fiber nano-probe. *Anal. Methods* **7,** 1307–1312 (2015).

[REF7] Leypold, C. F. Reiher, M. Brehm, G. Schmitt, M. O. Schneider, S. Matousek, P. & Towrie, M. Tetracycline and derivatives – assignment of IR and Raman spectra via DFT calculations. *Phys. Chem. Chem. Phys*. **5** 1149–1157 (2003).

[REF8] Chen, X. Liang, W. Yang, C. Lin, W., Bi, M. Simultaneous quantitative detection of tetracyclines derivates by Raman spectroscopy. In Proceedings of the 2012 IEEE International Conference on Virtual Environments Human-Computer

Interfaces and Measurement Systems (VECIMS) Proceedings, Tianjin, China, 2–4 July pp. 111–114 (2012).

[REF9] Lagergren, S. Zur theorie der sogenannten adsorption gelöster stoffe. Kungliga svenska vetenskapsakademiens. Kungliga svenska vetenskapsakademiens. *Handlingar* 1–39 (1889).

[REF10] Ho, Y. S. & McKay, G. Pseudo-second order model for sorption processes. *Process Biochem. Process Biochem*. **34**, 451–465 (1999).

[REF11] Chien, S. & Clayton, W. Application of Elovich equation to the kinetics of phosphate release and sorption in soils. *Soil Sci. Soc. Am. J.* **44**, 265–268 (1980).

[REF12] Blanchard, G., Maunaye, M., Martin, G. Removal of heavy metals from waters by means of natural zeolites. *Water Res.* **18** 1501-1507 (1984)

[REF13] Machado, F. M., Bergmann, C. P., Lima,c E. C., Royer, B., de Souza, F. E., Jauris, I. M., Calveted, T., Fagan, S. B. Adsorption of Reactive Blue 4 dye from water solutions by carbon nanotubes: experiment and theory. *Phys. Chem. Chem. Phys.* **14**, 11139-11153 (2012)

[REF 14] Roginsky, S., Zeldovich, Y.B. The catalytic oxidation of carbon monoxide on manganese dioxide. *Acta Phys. Chem. USSR* **1**, 554 (1934)

[REF 15] Lima, E. C., Adebayo, M. A., Machado, F. M. Kinetic and equilibrium models of adsorption, in Carbon nanomaterials as adsorbents for environmental and biological applications. *Springe*, pp. 33-69 (2015)
